# New Books

**Published:** 2010-12

**Climate Savvy: Adapting Conservation and Resource Management to a Changing World**

Lara J. Hansen, Jennifer R. Hoffman

Washington, DC:Island Press, 2010. 350 pp. ISBN: 978-1-59726-686-4, $40

**Coal and Peat Fires: A Global Perspective**

Glenn B. Stracher, Anupma Prakash, Ellina V. Sokol, eds.

St. Louis, MO:Elsevier, 2010. 380 pp. ISBN: 978-0-444-52858-2, $259.68

**Contesting the Future of Nuclear Power: A Critical Global Assessment of Atomic Energy**

Benjamin K Sovacool

Hackensack, NJ:World Scientific, 2011. 250 pp. ISBN: 978-981-4322-75-1, $60

**Database Technology for Life Sciences and Medicine**

Claudia Plant, Christian Böhm, eds.

Hackensack, NJ:World Scientific, 2010. 388 pp. ISBN: 978-981-4307-70-3, $108

**Energy in the 21st Century, 2nd ed.**

John R. Fanchi

Hackensack, NJ:World Scientific, 2010. 376 pp. ISBN: 978-981-4322-04-1, $120

**Evolution of Earth and its Climate: Birth, Life and Death of Earth**

O.G. Sorokhtin, G.V. Chilingarian and N.O. Sorokhtin

St. Louis, MO:Elsevier, 2010. 596 pp. ISBN: 978-0-444-53757-7, $170

**Facilitating Climate Change Responses**

Paul C. Stern, Roger E. Kasperson, eds.

Washington, DC:National Academies Press, 2010. 174 pp. ISBN: 978-0-309-16032-2, $37.25

**Modeling the Economics of Greenhouse Gas Mitigation**

K. John Holmes, ed.

Washington, DC:National Academies Press, 2010. 196 pp. ISBN: 978-0-309-16235-7, $44

**Monitoring Climate Change Impacts: Metrics at the Intersection of the Human and Earth Systems**

Committee on Indicators for Understanding Global Climate Change; National Research Council

Washington, DC:National Academies Press, 2010. 110 pp. ISBN: 978-0-309-15871-8, $29.25

**Review of the Proposal for the Gulf Long-Term Follow-Up Study**

Lynn Goldman, Abigail Mitchell, Margie Patlak, eds.

Washington, DC:National Academies Press, 2010. 30 pp. ISBN: 978-0-309-16244-9, $13.50

**Straight Up: America’s Fiercest Climate Blogger Takes on the Status Quo Media, Politicians, and Clean Energy Solutions**

Joseph J. Romm

Washington, DC:Island Press, 2010. 179 pp. ISBN: 978-1-59726-716-8, $21.96

**Unquenchable: America’s Water Crisis and What To Do About It**

Robert Glennon

Washington, DC:Island Press, 2010. 432 pp. ISBN: 978-1-59726-816-5, $19.95

